# Thyroid status influence on adiponectin, acylation stimulating protein (ASP) and complement C3 in hyperthyroid and hypothyroid subjects

**DOI:** 10.1186/1743-7075-3-13

**Published:** 2006-02-10

**Authors:** Haiying Yu, Yan Yang, Muxun Zhang, Huiling Lu, Jianhua Zhang, Hongwei Wang, Katherine Cianflone

**Affiliations:** 1 Department of Endocrinology, Tongji Hospital, Wuhan, Hubei, P.R. China; 2 Department of Pediatrics, Tongji Hospital, Wuhan, Hubei, P.R. China; 3 Centre de Recherche Hôpital Laval, Université Laval, Québec, Canada

## Abstract

**Background:**

Thyroid abnormalities (hyperthyroid and hypothyroid) are accompanied by changes in intermediary metabolism including alterations in body weight, insulin resistance and lipid profile. The aims of this study were to examine plasma ASP, its precursor C3 and adiponectin in hyperthyroid and hypothyroid subjects as compared to controls.

**Methods:**

A total of 99 subjects were recruited from endocrinology/out-patient clinics: 46 hyperthyroid subjects, 23 hypothyroid subjects and 30 control subjects. Subjects were evaluated for FT4, FT3, TSH, glucose, insulin, complete lipid profile and the adipokines: adiponectin, acylation stimulating protein (ASP) and complement C3.

**Results:**

Hyperthyroidism was associated with a 95% increase in adiponectin (p = 0.0002), a 47% decrease in C3 (p < 0.0001), no change in ASP and increased ASP/C3 ratio (p = 0.0012). Hypothyroidism was associated with a 31% increase in ASP (p = 0.008). Adiponectin and C3 correlated with FT3 (r = 0.383, p = 0.004 and r = -0.277, p = 0.007, respectively) and FT4 (r = 0.464, p = 0.003 and r = -0.225, p = 0.03, respectively). ASP correlated with TSH (r = 0.202, p = 0.04). Adiponectin did not correlate with either ASP or C3, only ASP and C3 correlated (r = -0.197, p = 0.05). Adiponectin was negatively correlated with BMI, total cholesterol and plasma triglyceride, while C3 was positively correlated with BMI and total cholesterol. Surprisingly, adiponectin was positively correlated with insulin (r = 0.293, p = 0.02) and HOMA-IR (r = 0.373, p = 0.003) while C3 was negatively correlated with glucose (r = -0.242, p = 0.022, insulin (r = -0.184, p = 0.05) and HOMA-IR.

**Conclusion:**

These changes suggest that thyroid disease may be accompanied by changes in adipokines, which may contribute to the phenotype expressed.

## Introduction

Thyroid abnormalities (hyperthyroid and hypothyroid) are accompanied by changes in intermediary metabolism including alterations in body weight, insulin resistance and lipid profile (review [1,2]. Specifically, hyperthyroidism is characterized by decreased body weight, increased insulin and glucose indicative of insulin resistance as well as decreases in plasma lipids such as plasma cholesterol and triglycerides. These changes are a consequence of the cellular effect of thyroid hormone on liver, muscle and adipose tissue metabolism, which leads to increased energy expenditure. Conversely, hypothyroidism is associated with decreased levels of triiodothyronine (T3), thyroxine (T4) and increased thyroid stimulating hormone (TSH). These changes lead to increased body weight with increased plasma lipids and lipoproteins. Interestingly, plasma triglyceride, LDL cholesterol and HDL cholesterol are all increased in hypothyroidism and decreased in hyperthyroidism [2,3], disrupting the normal inverse relationship seen between triglyceride and HDL cholesterol. As many of these changes are associated with alterations in glucose and insulin metabolism, and alterations in adipose tissue, we were interested in assessing the effect on adipokine hormones.

Adiponectin is a recently described adipose tissue hormone with multiple functions (review [4]). Plasma adiponectin has been reported to correlate with plasma lipids, in particular HDL cholesterol (HDL-C) (positively) [5,6], as well as to insulin resistance (negatively) with decreased plasma levels often reported in type 2 diabetes [7,8]. Interestingly, adiponectin, although produced in fat tissue, decreases in obesity [4]. Functional studies suggest that adiponectin administration can alter insulin sensitivity, but it can also mediate numerous anti-atherosclerotic vascular functions such as endothelial function, inhibition of smooth muscle cell proliferation and inhibition of macrophage conversion to foam cells (review [4]). Adiponectin has been suggested to increase fatty acid oxidation and alter energy expenditure [9,10], although this function remains controversial [11].

In contrast to the proposed effects of adiponectin on increasing energy expenditure, acylation stimulating protein (ASP) enhances energy storage through increasing postprandial triglyceride clearance, adipose tissue fatty acid esterification, glucose uptake and decreasing hormone sensitive lipase mediated lipolysis (review [12]). In a number of studies, ASP has been demonstrated to be increased in obesity, diabetes and cardiovascular disease (review [12]), and while not exclusively produced by adipose tissue, ASP generation increases in the adipose tissue milieu postprandially. Plasma ASP levels correlate positively with body mass index, as well as with plasma lipids such as total cholesterol, LDL cholesterol and apolipoprotein B. ASP often correlates with non-esterified fatty acids (NEFA)[12]. Interestingly, in C3(-/-) mice that are deficient in ASP, there is a decrease in adipose tissue mass with a repartitioning of ingested food towards increased energy expenditure (review [12]).

ASP, identical to C3adesArg, is produced through the alternate complement pathway via cleavage of complement C3, which is produced in multiple tissue sites, including the liver and adipose tissue. The cleavage of C3 is initiated through the action of adipsin (complement factor D), which, like adiponectin, is produced primarily in adipose tissue [13,14]. Independent of its role in generation of ASP, C3 appears to have direct links to insulin resistance and plasma lipids, an association that is specific to this complement protein [15,16]. C3 is increased in diabetes and in patients with coronary disease. C3 correlates strongly with insulin resistance [12], and in a study by Muscari *et al*, was shown to be a stronger predictor of insulin resistance and myocardial infarction than traditional risk factors [15].

The aims of this study were to examine plasma ASP, its precursor C3, and adiponectin in hyperthyroid and hypothyroid subjects as compared to controls. We tested (1) whether plasma ASP, C3 or adiponectin concentrations are associated with thyroid status in patients and (2) whether the levels of plasma C3 directly correlate with concentrations of ASP across all groups.

## Research design and methods

### Subjects

A total of 99 subjects were recruited from endocrinology and out-patient daily clinics at the Tongji Medical Centre, Tongji Hospital, Wuhan, Hubei, P.R. China. Participants ranged from 18 to 67 years old. All participants gave informed consent and the study was approved by Tongji Hospital Ethics Committee. Subjects with diabetes, liver disease, heart disease, infectious disease or any other known disease were excluded. All subjects were ambulatory and were studied as out-patients. No subjects were taking any medication or herbal remedies known to affect lipid metabolism. Control subjects were recruited during their evaluation for a regular medical check-up. All control subjects were non-obese and had T3, T4 and TSH values within normal ranges (see below).

All thyroid patients were evaluated at the time of diagnosis during their initial visit to the Endocrinology Clinic. Onset of symptoms was recent (within 2–3 months) and no patients were taking any thyroid medication or herbal remedies. Hyperthyroid subjects were primarily diagnosed with Graves disease, and presented with T3 and T4 above the normal ranges and TSH below the normal range (see below for ranges). Hypothyroid subjects were diagnosed with auto-immune disease, with biochemical parameters of T3 and T4 below normal ranges, and TSH above the normal range.

### Fasting blood samples and OGTT

Blood samples were drawn after an overnight fast from an antecubital vein. Fasting plasma samples were used for measurement of all parameters. Blood was centrifuged and plasma aliquoted and frozen immediately for future analysis of lipids and proteins.

### Analytical procedures

Blood glucose was determined by glucose-oxidase method (AVE-852 half-auto biochemical analyzer). Plasma NEFA concentration was determined by colorimetric enzymatic assay (WAKO Chemicals, Tokyo, Japan). Plasma triglycerides were measured by GPO-PAP method and total cholesterol was measured by COD-PAP method. Following precipitation of apoB containing lipoproteins, the concentration of HDL-cholesterol (HDL-C) was also measured by enzymatic colorimetric assay (BCR, Ai-Weihali autobiochemical analyzer). Inter and intra coefficient of variation (CV) for all parameters (except HDL) were <3%. For HDL, inter and intra CV were <5%.

FT3 (free T3), FT4 (free T4) and TSH were measured by automated chemiluminescence immunoassay (ACS-180, American Kang Ning Company). The intra assay and interassay coefficient of variation were 2% and 6% respectively, for both FT3 and FT4. The normal range within the clinical biochemistry laboratory for T3 is 3.95–6.47 pmol/L, for T4 the normal range is 11.99–21.00 pmol/L and for TSH the normal range is 0.27–4.2 mU/L. Insulin was measured by electrochemiluminescence immunoassay (Elecsys 1010, Roche Instrument Center AG). Plasma adiponectin concentration was measured by ELISA (B-Bridge International, Phoenix, AZ, USA). Complement C3 was measured by an immunoturbidimetric assay (Lin- Fei Co, P.R. China). Plasma ASP concentration was measured via a sandwich ELISA immunoassay as previously described in detail [17,18]. For these last assays, (insulin, adiponectin and ASP) intra-assay CV was <4% and interassay CV was <8%.

### Calculations

Body mass index (BMI) was calculated as weight (kg) per height (m^2^). Insulin resistance index was calculated by homeostasis model assessment, HOMA-IR, as (fasting insulin IU/L) * (fasting glucose mmol/L) / 22.5 as previously reported by Matthews [19]. LDL cholesterol (LDL-C) was calculated according to the Friedewald formula as LDL-C = (total cholesterol mmol/L) – (triglyceride mmol/L)/2.2 – (HDL-C mmol/L) [20].

### Statistical analyses

Unless otherwise stated, data are given as means ± standard error of the mean (SEM). Statistical comparisons of among all groups were performed using one-way ANOVA analyses. Correlations were analyzed with Pearson correlation coefficient or forward stepwise multiple regression analysis where *P <*0.05 was considered to be statistically significant for all analyses.

## Results

A total of 99 subjects were recruited: 46 hyperthyroid patients (13 men, 33 women), 23 hypothyroid patients (5 men, 18 women) and 30 control subjects (10 men, 20 women). Samples were taken at the time of the first visit to the out-patient/endocrinology clinic. Patients were excluded if they had heart disease, liver disease, diabetes or any other known disease including infectious disease. No subjects were taking any thyroid medication or any medication known to affect lipids or hormone levels (including any Chinese medicinal herbal preparations). Control subjects were healthy and non-obese, and all had normal levels of T3, T4 and TSH. Thyroid patients typically complained of palpitations, warmth and weight loss (hyperthyroidism) or cold sensitivity, fatigue and lethargy (hypothyroidism) but were generally healthy overall (see Methods for detailed information on hyperthyroid and hypothyroid classification). Onset of disease was recent (within 2–3 months). The clinical laboratory data are provided in Table 1. As shown in Table 1, the hyperthyroid (HYPER) group was slightly younger than the control group (CTL), but with similar body mass index (BMI). The hypothyroid (HYPO) group was matched for age, but had an increased BMI compared to the CTL group. Hyperthyroid subjects were characterized by increased fasting plasma glucose, insulin and HOMA-IR. As expected, based on diagnosis of hyperthyroidism, FT3 and FT4 were above normal, while TSH was below normal (vs. control subjects as indicated in Table 1). By contrast, in the HYPO group, glucose, insulin and HOMA-IR were comparable to CTL, but different from the hyperthyroid group. As expected, FT4 and FT3 were below normal, with a raised TSH level.

**Table 1:** Clinical and laboratory data in subject groups

Parameter	CTL	HYPER	HYPO	ANOVA
N	30	46	23	
Gender (M/F)	10/20	13/33	5/18	ns
Age (years)	41.7 ± 2.0	34.9 ± 1.6*	39.4 ± 2.5	0.029
BMI (kg/m^2^)	22.0 ± 0.4	21.2 ± 0.4^†††^	24.2 ± 0.61*	0.0003
Glucose (mmol/L)	4.88 ± 0.11	5.47 ± 0.07***	5.08 ± 0.12^†^	<0.0001
Insulin (pmol/L)	34.0 ± 5.5	60.8 ± 4.9***	33.8 ± 2.8^††^	<0.0001
HOMA-IR	1.10 ± 0.20	1.86 ± 0.16**	1.02 ± 0.11^††^	0.0004
FT4 (pmol/L)	17.4 ± 0.4	64.5 ± 8.4***	9.1 ± 0.6*^†††^	<0.0001
FT3 (pmol/L)	5.7 ± 0.1	13.3 ± 1.2***	3.2 ± 0.2**^†††^	<0.0001
TSH (mU/L)	2.1 ± 0.1	0.03 ± 0.02***	69.5 ± 21.1**^†††^	<0.0001

Results are expressed as average ± SEM for the groups indicated as control (CTL), hyperthyroid (HYPER) and hypothyroid (HYPO) where N = number of subjects per group. Significance was calculated by ANOVA with Bonferroni post-hoc test. Differences vs. control (CTL) group are indicated as *P < 0.05,**P < 0.01,***P < 0.001; and differences for hyperthyroid vs. hypothyroid as ^†^p < 0.05, ^††^p < 0.01, ^†††^p < 0.001, ns = not significant.

Fasting lipid profiles are shown in Table 2. In the HYPER group, plasma triglycerides, total cholesterol, LDL cholesterol and HDL cholesterol were all strikingly reduced compared to the CTL group, whereas NEFA concentrations were substantially increased. By contrast, in the HYPO group, plasma triglycerides, total cholesterol and LDL cholesterol were all substantially increased compared to CTL.

**Table 2:** Fasting lipid profiles in subject groups

Parameter	CTL	HYPER	HYPO	ANOVA
N	30	46	23	<0.0001
TG (mmol/L)	1.14 ± 0.12	0.73 ± 0.06**,^†††^	1.74 ± 0.09***	<0.0001
TChol (mmol/L)	4.58 ± 0.15	2.72 ± 0.14***,^†††^	6.16 ± 0.24***	<0.0001
LDL-C (mmol/L)	2.40 ± 0.16	1.41 ± 0.09***,^†††^	3.66 ± 0.18***	<0.0001
HDL-C (mmol/L)	1.56 ± 0.06	0.99 ± 0.04***	1.74 ± 0.07^†††^	<0.0001
NEFA (mmol/L)	0.361 ± 0.03	0.617 ± 0.05***	0.425 ± 0.03^†††^	<0.0001

Results are expressed as average ± SEM for the four groups indicated as control (CTL), hyperthyroid (HYPER) and hypothyroid (HYPO) where TG = plasma triglyceride, TChol = total cholesterol, LDL-C = LDL cholesterol, HDL-C = HDL cholesterol and NEFA = non-esterified fatty acids. Significance was calculated by ANOVA with Bonferroni post-hoc test where differences vs. control (CTL) group are indicated as *P < 0.05,**P < 0.01,***P < 0.001; and differences between hyperthyroid and hypothyroid are indicated as ^†^p < 0.05, ^††^p < 0.01, ^†††^p < 0.001.

**Figure 1:**
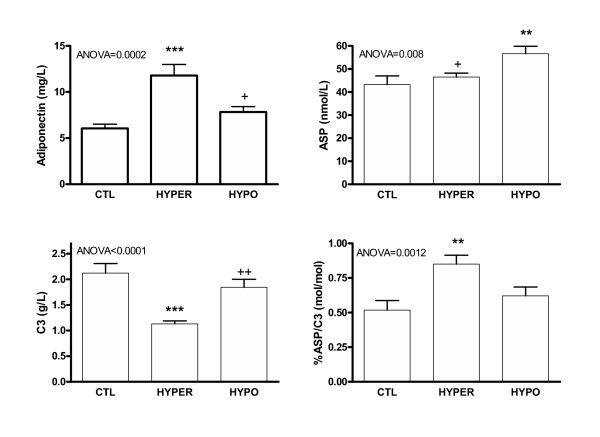
**Adipocyte hormones in control (CTL), hyperthyroid (HYPER) and hypothyroid (HYPO) subjects**. Results are expressed as average ± SEM for the three groups indicated. Significance was calculated by ANOVA for all groups with Bonferroni post-hoc test where differences vs. control (CTL) group are indicated as *P < 0.01,***P < 0.001; and differences for HYPO vs. HYPER indicated as ^+^p < 0.05 and ^++ ^p < 0.01.

The results for adipose tissue hormones are shown in Figure 1. In the HYPER group, adiponectin was substantially increased by 95% as compared to CTL group (p = 0.0002 by ANOVA), while C3 was decreased by 47% (p < 0.0001 by ANOVA). Similar differences were obtained for adiponectin when separated into male and female groups (not shown). In spite of the substantial reduction in C3, the precursor to ASP, ASP levels remained normal, therefore the %ASP/C3 ratio increased by 64% (p = 0.0012 by ANOVA). By contrast, in the HYPO group, there was no significant change in either adiponectin or C3 compared to CTL, but there was an increase in ASP of 31% (p = 0.008 by ANOVA).

As shown in Table 3, all three adipose tissue hormones and insulin correlated significantly with indices of thyroid function. Specifically, adiponectin and C3 correlated with FT3 (r = 0.382, p = 0.002 and r = -0.278, p = 0.007, respectively) and FT4 (r = 0.464, p = 0.0001 and r = -0.225, p = 0.03, respectively). Insulin also correlated with both FT3 and FT4 (r = 0.389, p = 0.003 and r = 0.395, p = 0.003, respectively). ASP correlated with TSH (r = 0.202, p = 0.04). Adiponectin did not correlate with either ASP or C3, only ASP and C3 correlated (r = -0.197, p = 0.05).

Further both C3 and adiponectin correlated with a number of plasma parameters. Adiponectin was negatively correlated with BMI, total cholesterol and plasma triglyceride, while C3 was positively correlated with BMI and total cholesterol. Surprisingly, adiponectin was positively correlated with insulin and HOMA-IR, while C3 was negatively correlated with glucose, insulin and HOMA-IR. In best subset regression analysis, adiponectin was best predicted by a combination of: FT4 (p = 0.001) + HOMA-IR (p = 0.05) + TChol + HDL-C + LDL-C (p = 0.05) (r^2 ^= 0.368, p = 0.001). Obligatory inclusion of BMI did not improve the correlation. C3 was best predicted by a combination of ASP, NEFA and HDL (r^2 ^= 0.369 where ASP p = 0.012, NEFA p = 0.031 and HDL-C p = 0.042). Obligatory inclusion of BMI did not improve the correlation. Finally, ASP was best predicted by a combination of TSH, C3, NEFA, BMI, TChol, HDL-C and LDL-C (r^2 ^= 0.240, with the following individual p values: TSH (0.012), C3 (0.018), BMI (0.050), TChol (0.048), HDL-C (0.063).

**Table 3:** Correlations between thyroid hormones, adipokines and plasma parameters

Parameter	Adiponectin	ASP	C3	%ASP/C3	Insulin
	r/(P)	r/(P)	r/(P)	r/P	r/(P)
FT3	0.382(.002)	-.022(.831)	-0.278(.007)	0.222(.030)	0.389(.003)
FT4	0.464(.0001)	0.020(.851)	-0.225(.03)	0.147(.158)	0.395(.003)
TSH	-0.080(.538)	0.202(.04)	0.121(.252)	-0.022(.834)	-0.071(.497)
BMI	-0.220(.05)	-0.07(.445)	0.186(.05)	-0.181(.05)	-0.047(.653)
Glucose	0.230(.07)	-0.022(.836)	-0.242(.022)	0.273(.009)	0.250(.018)
Insulin	0.293(.02)	0.114(.273)	-0.184(.05)	0.175(.05)	----------
HOMA-IR	0.373(.003)	0.132(.224)	-0.184(.05)	0.174(.103)	----------
TChol	-0.270(.032)	0.105(.307)	0.330(.001)	-0.233(.022)	-0.253(.013)
TG	-0.319(.012)	0.108(.291)	0.162(.123)	-0.104(.320)	-0.061(.561)
HDL-C	-0.147(.249)	0.035(.731)	0.417(.00002)	-0.350(.00005)	-0.115(.265)
LDL-C	-0.195(.128)	0.099(.336)	0.291(.004)	-0.218(.034)	-0.287(.004)
NEFA	0.110(.397)	0.015(.885)	-0.251(.015)	0.225(.028)	0.029(.776)

Results are provided for correlations (with P values indicated) for the whole cohort of subjects where BMI = body mass index, TChol = total cholesterol, TG = plasma triglyceride, NEFA = non-esterified fatty acid. Correlations which are significant are underlined.

## Discussion

The salient findings in the present study are the association of hyperthyroidism with increased adiponectin, decreased C3 and increased ASP/C3 ratio, and of hypothyroidism with increased ASP. Further, these changes in adipokines are each associated with specific changes in glucose or lipid metabolism which reflect whole body changes in energy expenditure/energy storage as discussed below. Thus this study provides important new data in this area.

With respect to adiponectin, the increased levels in hyperthyroidism are consistent with the increased metabolic rate that is characteristic of these patients. Our results contrast with those of Iglesias [21], where no significant changes were found. However it is difficult to draw conclusions since the average levels of adiponectin in that study were greater in all groups with a large range in values obtained (based on reported s.e.m.), the immunoassays used were different from the present study, and the age of the subjects was older. A study by Santini *et al *also examined adiponectin in hyperthyroid/hypothyroid subjects and found no significant differences. However the subject size was small, and this may have confounded the data (the authors suggest this to explain the lack of correlation in their study between adiponectin and BMI) [22]. In contrast with these two studies, Yaturu and colleagues did find that adiponectin levels were higher in the hyperthyroid state than in the hypothyroid state, in agreement with our study [23]. Further, a study of 68 healthy subjects demonstrated a positive association between plasma adiponectin and thyroid hormones although hyperthyroid and hypothyroid subjects were not tested directly [24].

Interestingly, in our study, the hyperthyroid subjects are also insulin resistant, with increased insulin levels. While insulin resistance is usually associated with decreased adiponectin levels, at least in diabetics [4,7,8], in hyperthyroidism this inverse relationship appears to be lost, and there is a direct correlation of adiponectin with plasma insulin and HOMA-IR. This is also true of the adiponectin association with plasma lipids. While several studies in controls, diabetic subjects and patients with cardiovascular disease have demonstrated a positive correlation of adiponectin with HDL-C [5,6,25-30], this is not true in the hyper/hypo thyroid subjects. In fact adiponectin correlates inversely with total plasma cholesterol and triglycerides but not HDL-C. This likely relates to the disruption of the usual elevated triglyceride/low HDL cholesterol relationship, which is a feature of hyper/hypothyroidism [2,3]. This association of increased adiponectin with decreased plasma lipids but increased NEFA is consistent with an implication of adiponectin in fatty acid metabolism. Enhanced release of NEFA from adipose stores, coupled to increased adiponectin may stimulate fatty acid oxidation in muscle and liver [9] with a comparable decrease in lipoprotein production [10]. The result is an anti-atherogenic lipid profile, which may be mediated through both thyroid hormones as well as adiponectin. This raises some interesting questions/hypotheses as to the mechanism of increased adiponectin secretion from adipocytes in hyperthyroid subjects. Evidence for a role of thyroid hormones in the regulation of adiponectin expression is suggested in a recent study showing increased adiponectin in mice exposed to cold [31]. On the other hand, insulin has also been reported to stimulate adiponectin secretion [32-34], and the increased levels in the hyperthyroid subjects may also act as a stimulus. These hypotheses, however, remain to be tested experimentally.

In contrast to adiponectin, C3 was markedly decreased in hyperthyroid subjects, while ASP was increased in hypothyroid patients as compared to CTL. Directionally, the differences between hyper- and hypo- thyroid patients were the same for ASP and its precursor C3, in that both C3 and ASP were greater in hypothyroid than in hyperthyroid. However, the correlation of C3 with lipid and glucose parameters was stronger than the correlations with ASP. Other than the requirement of C3 as precursor for ASP, there is no known role for C3 in glucose or lipid metabolism, notwithstanding the many studies that have documented correlations of C3 with glucose and lipid metabolism and insulin resistance (review [12]. This raises the question: Is there a direct effect of thyroid hormones on C3 production? Thyroid hormones bind to TREs (thyroid response elements), which heterodimerize with RAR and RXR (retinoic acid response elements) [35,36], the latter of which is known to regulate C3 expression [37], explaining the close correlation with C3. Alternately, do the changes in thyroid hormones increase C3 conversion to ASP or ASP clearance rates? How thyroid hormones affect this process remains to be seen.

While this is the first report of ASP in hypothyroidism, the increased plasma levels of ASP are certainly consistent with the increased BMI and lipids. As detailed elsewhere (review [12], increased ASP is associated with obesity, diabetes and increased fasting plasma cholesterol, triglyceride, apolipoprotein B and NEFA. Further, in oral fat load studies, a higher ASP is associated with both increased fasting triglyceride and a delay in triglyceride clearance, suggesting ASP resistance [38]. While we can only speculate, this may also be true of hypothyroid subjects. True to its proposed metabolic function, the increased plasma ASP levels are indicative of a push towards NEFA storage rather than oxidation, towards decreased energy metabolism, and towards an increase rather than a decrease in BMI. Interestingly in this setting of increased lipids but normal glucose, only ASP is increased while insulin remains at normal levels. These results dovetail with the studies on C3(-/-) ASP deficient mice, which are deficient in ASP and manifest increased energy expenditure with decreased body fat [39-41], while in the present subjects a decrease in energy expenditure coupled with an increase in BMI are associated with increased plasma ASP.

While, as noted above, there is no information available on the direct effects of thyroid hormones on adiponectin, ASP or C3 synthesis or secretion, there is additional information on other cytokines in thyroid dysfunction. IL-6 and TNFα are both increased in Grave's disease (hyperthyroid state) [42,43], however since these cytokines have been reported to decrease adiponectin [44] and increase C3 [45], this cannot explain the demonstrated increase in adiponectin and decrease in C3 in the hyperthyroid state in the present study. However there is no lack of information on the thyroid hormone mediated effects on adipose tissue *in vitro *and *in vivo*. Thyroid hormone increases adipose tissue lipolysis as measured by increased NEFA or glycerol release [46-48] and increased β adrenergic response [49-51]. Contrasting with the push for lipolysis, hyperthyroidism induces down regulation of adipose fatty acid synthase and lipoprotein lipase; most of these effects reversed in hypothyroidism [52-54].

In summary, adipokines likely play an important role in mediating energy partitioning towards either utilization or storage. In that respect, ASP and adiponectin appear to have opposite metabolic effects. In hyperthyroid subjects, adiponectin is increased in a state where there is increased fatty acid oxidation, whereas in hypothyroidism ASP is increased where there is a push towards energy storage. Thus the well documented effects of thyroid hormone stimulation of energy expenditure in human and animal studies may partly be achieved through enabling effects on adipokines.

## Abbreviations

ASP: acylation stimulating protein; BMI: body mass index; C3: complement C3; HDL-C: high density lipoprotein cholesterol; HOMA-IR: homeostasis model assessment of insulin resistance; LDL-C: low density lipoprotein cholesterol; NEFA: non-esterified fatty acids; PPARγ: peroxisome proliferator-activated receptor γ; TG: triglyceride.
